# Elaboration of a nomogram to predict nonsentinel node status in breast cancer patients with positive sentinel node, intraoperatively assessed with one step nucleic amplification: Retrospective and validation phase

**DOI:** 10.1186/s13046-016-0460-6

**Published:** 2016-12-08

**Authors:** Franco Di Filippo, Simona Di Filippo, Anna Maria Ferrari, Raffaele Antonetti, Alessandro Battaglia, Francesca Becherini, Laia Bernet, Renzo Boldorini, Catherine Bouteille, Simonetta Buglioni, Paolo Burelli, Rafael Cano, Vincenzo Canzonieri, Pierluigi Chiodera, Alfredo Cirilli, Luigi Coppola, Stefano Drago, Luca Di Tommaso, Privato Fenaroli, Roberto Franchini, Andrea Gianatti, Diana Giannarelli, Carmela Giardina, Florence Godey, Massimo M. Grassi, Giuseppe B. Grassi, Siobhan Laws, Samuele Massarut, Giuseppe Naccarato, Maria Iole Natalicchio, Sergio Orefice, Fabrizio Palmieri, Tiziana Perin, Manuela Roncella, Massimo G. Roncalli, Antonio Rulli, Angelo Sidoni, Corrado Tinterri, Maria C. Truglia, Isabella Sperduti

**Affiliations:** 1 Regina Elena National Cancer Institute, Via Elio Chianesi 53, 00144 Rome, Italy; 2 Ospedale di Latina, Latina, Italy; 3 San Camillo, Milan, Italy; 4 Az. Ospedaliera Universitaria Foggia, Foggia, Italy; 5 ASL, Prato, Italy; 6 ULSS 7 Pieve di Soligo, Pieve di Soligo, Italy; 7 Hospital Lluís Alcanyís, Xàtiva, Spain; 8 Università of Piemonte, Vercelli, Italy; 9 Hôpitaux de Lyon, Lyon, France; 10 Hospital Universitario de La Ribera, Alzira, Spain; 11 Centro Regionale Oncologico, Bari, Italy; 12 San Donato, Italy; 13 Policlinico of Bari, Bari, Italy; 14 San Filippo Neri, Florence, Italy; 15 Humanitas Rozzano, Rozzano, Italy; 16 ASST Papa Giovanni XXIII, Bergamo, Italy; 17 Azienda Ospedaliera “Maggiore della Carità” di Novara, Novara, Italy; 18 University of Bari, Bari, Italy; 19 Eugene Marquis Cancer Center, Rennes, France; 20 Humanitas Gavazzeni, Bergamo, Italy; 21 Hampshire Hospitals NHS Foundation Trust, England, UK; 22 University of Pisa, Pisa, Italy; 23 Azienda Ospedaliero-Universitaria OO.RR. Foggia, Foggia, Italy; 24 University of L’Aquila, L’Aquila, Italy; 25 I.R.C.C.S. L. Spallanzani, Rome, Italy; 26 Azienda Ospedaliero-Universitaria Pisana, Pisa, Italy; 27 Humanitas, Pisa, Italy; 28 University of Perugia, Perugia, Italy; 29 USL, Prato, Italy

**Keywords:** Nomogram, Non Sentinel Node status, OSNA method, CK19 mRNA number copies

## Abstract

**Background:**

Tumor-positive sentinel lymph node (SLN) biopsy results in a risk of non sentinel node metastases in micro- and macro-metastases ranging from 20 to 50%, respectively. Therefore, most patients underwent unnecessary axillary lymph node dissections. We have previously developed a mathematical model for predicting patient-specific risk of non sentinel node (NSN) metastases based on 2460 patients. The study reports the results of the validation phase where a total of 1945 patients were enrolled, aimed at identifying a tool that gives the possibility to the surgeon to choose intraoperatively whether to perform or not axillary lymph node dissection (ALND).

**Methods:**

The following parameters were recorded: Clinical: hospital, age, medical record number; Bio pathological: Tumor (T) size stratified in quartiles, grading (G), histologic type, lymphatic/vascular invasion (LVI), ER-PR status, Ki 67, molecular classification (Luminal A, Luminal B, HER-2 Like, Triple negative); Sentinel and non-sentinel node related: Number of NSNs removed, number of positive NSNs, cytokeratin 19 (CK19) mRNA copy number of positive sentinel nodes stratified in quartiles. A total of 1945 patients were included in the database. All patient data were provided by the authors of this paper.

**Results:**

The discrimination of the model quantified with the area under the receiver operating characteristics (ROC) curve (AUC), was 0.65 and 0.71 in the validation and retrospective phase, respectively. The calibration determines the distance between predicted outcome and actual outcome. The mean difference between predicted/observed was 2.3 and 6.3% in the retrospective and in the validation phase, respectively. The two values are quite similar and as a result we can conclude that the nomogram effectiveness was validated. Moreover, the ROC curve identified in the risk category of 31% of positive NSNs, the best compromise between false negative and positive rates i.e. when ALND is unnecessary (<31%) or recommended (>31%).

**Conclusions:**

The results of the study confirm that OSNA nomogram may help surgeons make an intraoperative decision on whether to perform ALND or not in case of positive sentinel nodes, and the patient to accept this decision based on a reliable estimation on the true percentage of NSN involvement. The use of this nomogram achieves two main gools: 1) the choice of the right treatment during the operation, 2) to avoid for the patient a second surgery procedure.

## Background

In the treatment of breast cancer patients sentinel lymph node (SLN) biopsy is a highly accurate predictor of overall axillary status. It has become the standard axillary staging method for the last 15 years in breast cancer (BC) patients who are confirmed clinically negative for lymph node metastases [1, 2]. In the case of negative SLN, patients can safely avoid axillary lymph node dissection (ALND), thus preventing associated morbidity [3]. However, approximately 50–70% of patients with positive SLN have no additional positive nodes, suggesting that it may be possible to avoid ALND in selected patients [4, 5]. Taking these considerations into account, an accurate estimate of the likelihood of additional node metastases may be of paramount importance when deciding further treatment. At present, the intra-operative decision on, whether to perform ALND or not, is often only based on the positivity of the SLN. In order to assess the SLN status more rapidly, a semi-automated molecular method called the one step nucleic acid amplification (OSNA) assay has recently been made available [6, 7]. As a matter of fact this method is able to assess the entire SLN in thirty minutes. On the basis of these considerations, the European OSNA Committee decided to develop a new nomogram able to predict the non sentinel node (NSN) status, aimed at identifying a tool that gives the possibility to the surgeon to choose intraoperativily whether to perform or not axillary lymph node dissection (ALND). A total of 2460 patients were enrolled in the retrospective phase of the nomogram elaboration. The multivariate analysis demonstrated that only the number of CK19 copies (*p* < 0.0001) and T size (*p* < 0.0001) were associated with the NSN metastases. Therefore, a nomogram was developed using these two parameters stratified in quartiles. The score of each of the two variables summed and reported in on the total raw score immediately below the percentage of NSN positivity is identified [8]. The aim of the study was to report the results of the validation phase comprising a total of 1495 enrolled patients (the retrospective phase was already published). The study was conducted with the support of 22 European centers that did not requested any financial support.

## Methods

### Patients’ population

The European OSNA Users Committee decided to carry out the validation phase of the nomogram project with the following aims: To verify the effectiveness of the nomogram to help surgeons in deciding whether to carry out ALND in case of positive SLN; to identify patients at very low risk of positive NSNs in which ALND may be avoided. Our study population only included cases that fulfilled the following criteria: primary invasive cT1-3 BC with clinically and radiological (preoperative sonogram) negative axilla; no prior systemic treatment, or axillary surgery; successful SLN biopsy in which metastatic disease was identified by OSNA; and ALND with at least 10 nodes examined. The following parameters were recorded: Clinical: hospital, age, medical record number; Bio-pathological: tumor size stratified in quartiles, grading, multifocality, histological type, LVI, ER-PR status, HER-2, ki67, molecular classification (luminal A, luminal B, HER2 like, triple negative); SLN and NSN related: number of removed SLNs, number of positive and negative SLNs, copy number of positive SLNs. A total of 2460 patients were included in the database in the retrospective phase. Seventeen European centers contributed in the retrospective enrollment of patients in the validation phase up to a total of 1495 patients.

The biopathological parameters and the characteristics of SN and NSN are shown in Tables 1 and 2.

**Table 1:** Clinicopathological characteristics of patients

Characteristics	N of patients	Percent
Histology		
IDL	1278	85.5
ILC	184	12.3
Other	33	2.2
Grading		
G1	129	8.6
G2	827	55.4
G3	490	32.8
Unk	49	3.2
ER		
Pos	1297	86.7
Neg	147	9.8
Unk	51	3.4
PgR		
Pos	1174	78.5
Neg	268	17.9
Unk	53	3.5
HER2		
Pos	147	9.8
Neg	927	62.0
Unk	421	28.2
Ki67		
Low	876	58.6
High	515	34.4
Unk	104	7.0
T		
≤ 12	398	26.6
≥ 13–18	364	24.3
≥ 19–25	400	26.8
> 25	333	22.3
Type		
Multiple	364	24.3
Single	1131	75.7
Molecular subtype		
Luminal A	452	30.2
Luminal B	628	42.0
HER2-like	50	3.3
Triple Negative	58	3.9
Unk	307	20.5

**Table 2:** Characteristics of non sentinel node and sentinel node

	Number	Percent
NSLNs Examined		
Median (range)	15 (11–52)	
N° of positive NSLNs	610	40.8
Median (range)	2 (1–41)	
N° of Copies (Highest copy number)		
≤ 1500	305	20.4
> 1500–12,000	329	22.0
> 12,000–111,000	460	30.8
> 111,000	401	26.8

LVI was excluded because the aim of the nomogram is to give the possibility to the surgeon to intra operatively establish whether to perform ALND or not and this parameter cannot be assessed reliably in the preoperative breast cancer biopsy.

### Sentinel Lymph Node (SLN) sampling method

SLNs were identified using technetium 99 m- labeled, nanosized, human serum albumin colloids. To avoid any contamination during tumor manipulation, SLNs were surgically excised before breast surgery and sent on ice to the Pathology Department. Each SLN was weighed and measured. SLNs weighing less than 50 mg were excluded from the study. SLNs weighing more than 600 mg were cut in two or more pieces and processed as separate nodes. The weight of lymph node for homogenization should be within a range of 50/600 mg. If the weight of the lymph node is either above or below this specified range accurate results may not been obtained.

### One Step Nucleic Acid Amplification (OSNA)

The OSNA assay was performed according to the manufacturer’s instructions (Sysmex, Kobe, Japan). In short, the SLN was homogenized in 4 ml of the LINORHAG homogenizing buffer (Sysmex) on ice. A small aliquot was used for automated real-time amplification of CK19 mRNA via reverse transcription loop-mediated isothermal amplification (RT-LAMP) with the ready-to use LYNOAMP reagent kit (Sysmex) on the RD-100i (Sysmex). It was possible to analyze up to 4 SLNs in one run. The degree of amplification was detected via a byproduct of the reaction, i.e. magnesium-pyrophosphate. After use, the excess lysate was stored at minus 80 °C. A lysate with CK19 mRNA copy number/μl less than 250 (a) was regarded as negative (score−); from 250 to 5000 (b) as positive (score +), and greater than 5000 (c) (score ++). The OSNA results were immediately communicated to the surgeon by telephone within 30–40 min. For statistical analysis, in case of two or more SLNs, the SLN with the greatest CK19 mRNA copies was chosen. When there was a positive OSNA result, both for micro-metastases (+) and macro-metastases (++), the patients underwent an immediate ALND. ITCs are not detected by the OSNA method. This is not a limitation because patients with positive SLNs for ITC are no longer referred to undergo ALND.

Axillary NSNs were routinely examined by H&E.

### Statistical method

The outcome of our nomogram was the presence of positive nodes in the axillary dissections following OSNA evaluation in the population defined above. In order to validate the retrospective phase of the nomogram we have considered the covariates that predicted this outcome in the previous published paper [8], thus the endpoint was a binary outcome (presence versus absence of at least one positive node other than SLN) and the association with the covariates was analyzed using a logistic linear model. Discrimination ability was assessed by ROC analysis and predictive accuracy was measured by the AUC reported with its 95% confidence interval. Calibration was evaluated by reviewing the plot of predicted probabilities versus the actual probabilities. Well calibrated models have a linear relationship with a slope of 1 and an intercept of 0. Thus, a linear regression coefficient between predicted and observed values was estimated. The resulting model will be validated in a prospective series. All the analyses were performed using IBM SPSS version n. 20 [9].

### Ethical consideration

Patient data was anonymously gathered retrospectively with no influence on patient therapy. The Nomogram project was approved by the Ethics Committee of each participating institute.

## Results

**Fig. 1:**
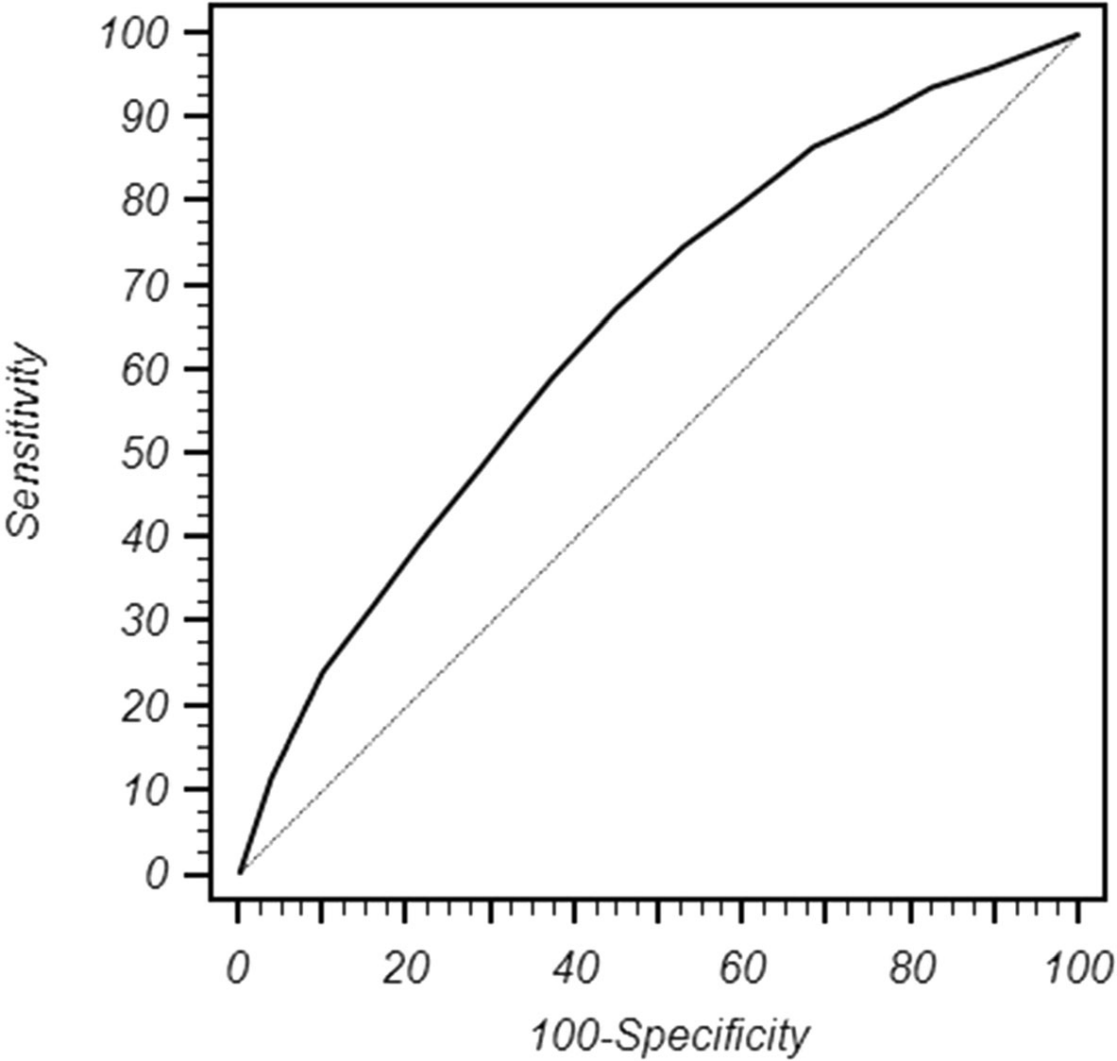
ROC curve of of number of CK19 mRNA, T size (quartiles) and the model containing these two variables

**Fig. 2:**
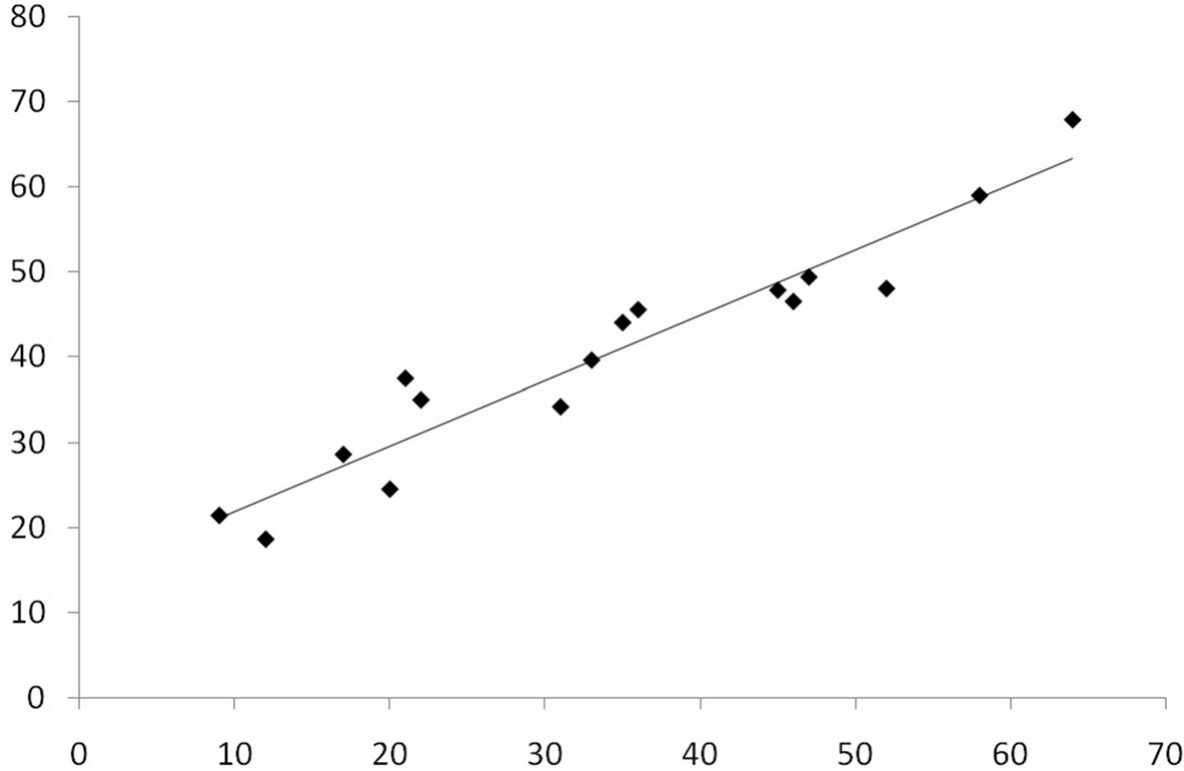
The model performs well and correctly at low and in high risk as shown in calibration plot. The linear regression model has a slope of 0.96 (95% C.I. 0.98/1.40) and a constant of -13.8 between predicted and actual probabilities (95%C.I. -22.9/-4.85)

Table 1 shows the clinical and bio-pathological characteristics of the patients. The mean and median ages were 55 and 54, respectively and standard deviation was 13 and range 24–80 years. The vast majority of the patients were affected with infiltrating ductal carcinoma (85.5%). Most of them had an intermediate (55.4%) or high grade tumors (32.8%). Both Estrogen (ER) and Progesterone (PgR) receptors were positive in 86.7 and 78.5%, respectively, whereas HER2 was positive only in 9.8% of the patients. Ki67 was high in 34.4% and LVI was present in 24.2% of the patients. These parameters represent the new molecular classifications of breast cancer that not only allow to identify patients at a higher risk of relapse but may also guide postoperative therapies [10, 11]. Tumor size was divided in quartiles, the cut-offs being 12, 18 and 25 mm. The mean and median tumor sizes were 20.5 and 18.2 mm, respectively, ranging between 0.7 and 50 mm. The SLNs and NSLNs characteristics are reported in Table 2. The median number of NSNs removed with ALND is 15 (range11–52). The number of positive NSNs was 610 (40.8%), the median value was 2 (range 1–41). The number of CK19 mRNA was divided in quartiles in order to obtain a better stratification of the patients. In order to validate the nomogram, we evaluated the discrimination of the model. This parameter which was quantified with the area under receiver operating characteristic (ROC) curve was 0.65 (95% C.I. 0.63–0.69). Figure 1 shows the ROC curve of the validation phase, the values are quite similar being 0.71 in the retrospective phase and 0.65 in the validation phase, demonstrating a fair level of discrimination. Another parameter that is usually employed to evaluate the reliability of a nomogram is the calibration, that is shown in Fig. 2. The calibration determines the distance between predicted outcome and actual outcome. The mean difference between predicted/observed was 2.3 and 6.3% in the retrospective and in the validation phase, respectively. The two values are quite similar, and consequently we can conclude that the nomogram effectiveness was validated [8].

It is well known that SLN micro- and macro-metastases are associated with a mean NSN positivity rate of 20 and 50%, respectively. Consequently, the dilemma for surgeons still persists in how to avoid unnecessary ALND and how to identify patients at high risk of positive NSNs in which ALND is recommended. Given that the validation phase had been evaluated successfully, all the patients enrolled in the retrospective and validation phase (3955 patients were valuable for the nomogram) were evaluated to develop a tool that allows the surgeon to intraoperatively make a decision on whether to perform ALND in case of positive SLN or not. The nomogram validated the risk percentage of NSN positivity that we recently published [8]. In Table 3, we stratified the patients in risk categories, according the nomogram model, from 11 to 50% and for each risk category we calculated the percentage of false negative and false positive rates in order to identify patients in which ALND is unnecessary and those where ALND is recommended. Moreover, the ROC curve identified in the risk category of 31%, the best compromise between false negative and positive rates. Therefore, in patients below this cut-off ALND may be omitted, for values higher than 31% ALND is recommended.

**Table 3:** Percentage of FN and FP rates according to value risk categories are reported

Risk Categories	11%	20%	30%	45%	50%	31%
% False negative (FN)	1%	3.5%	8.2%	18.4%	24.4%	8.9%
% False positive FP)	56.0%	45.6%	31.4%	15.6%	9.0%	30.1%

## Discussion

Usually the effectiveness of a nomogram is evaluated with three parameters i.e., discrimination, calibration and the capacity of a nomogram to identify false negatives i.e. patients with a risk of NSN metastases ≤10% in which ALND may be omitted. Discrimination (i.e. whether the relative ranking of individual prediction is in the correct order) was quantified with the area under curve the receiver operating characteristics (ROC) curve (AUC). The AUC is a summary measure of the ROC that reflects the ability of a test to discriminate between a diseased and non-diseased subject across all the possible levels of positivity. AUC ranges from 0 to 1, with 1 indicating perfect concordance, 0.5 indicating no better concordance than “flip the coin”, and indicating perfect discordance.

In our nomogram, the AUCs are 0.71 and 0.65, respectively that are considered a fair value of discrimination consistent with the best nomograms published so far. This data has been confirmed by a recent publication by Van Den Hoven who reported a “Head to Head” comparison of nine predictive tools [12]. The majority of nomograms include tumor size, lymphovascular invasion, and the size of the SLN metastases. This is very consistent with our predictive tools in which the multivariate analysis selected T-size, number of mRNA copies in the SLN (i.e.) and tumor load. The comparison of nine predictive tools showed that the MSKCC nomogram had best discrimination with an AUC of 0.69, followed by the Stanford, Mayo and MOU models with AUC’s of 0.66, 0.65 and 0.65, respectively. The Stanford model has second best discrimination (AUC 0.66) and the Mayo and MOU models are tied for third (AUC 0.65). These data confirm that the AUC’s of both retrospective and validation phases are perfectly consistent with the best nomograms published so far. Calibration determines how far the predicted probabilities are from the actual outcomes, that has a higher clinical significance than discrimination. Recently, Coutant evaluated the AUC and calibration of 9 previously published predicted models [13]. Coutant found that two of the nomograms were well calibrated, whereas the other two showed differences between predicted and observed probabilities. It was also outlined that the difference between the predicted and observed probabilities for these nomograms range from 3 to 25%. In our nomogram, the values were 2.3 and 6.3% for retrospective and validation phase, respectively. Therefore, they belong to the category of low values of the above mentioned range and can be considered reliable. This information is of clinical utility because it gives clinicians the opportunity to inform patients about the predicted probability of NSN metastases. As far as the false negative rate is concerned, we stratified our patients in risk categories in a range from 11 to 50% and the percentage of false negative and positive rates was calculated for each risk category (Table 3). It is readily apparent that up to risk category of 30% the false negative rate is 8.2% that is in the range value of the false negative rate reported for SLNB technique. Therefore, up to this category surgeons may feel comfortable in suggesting patients to not undergo ALND and patients accepting this decision. It is worth considering that nomograms safely avoided ALNDs in 1254 (32%). We used the ROC curve analysis to calculate best level of risk category in terms of balance between false positive and negative rates. The ROC curve identified a risk category of 31% as the optimal cut-off that the surgeon may employ in the decision-making process on whether ALND may be omitted (<31%) or recommended (>31%). This value was calculated taking into account the sensitivity and specificity of the cut-off. If we would have chosen other cut-offs this would result in a decrease of sensitivity or specificity, therefore this topic has to be discussed when counseling the patients. In fact in the risk category of 30% the false negative rate is 8.2% which is acceptable. These considerations are to be considered valid because in the risk categories >31% we verified patients with a percentage of positive NSNs of 60.2%, 63% (>45%) and 66.5% (>50%). Moreover, in the vast majority of these patients there were more than 3 positive NSNs. In our previous paper we evaluated the capacity of the nomogram in identifying how many false negative patients were in the risk category of 10% only because this parameter (together wih discrimination and calibration) is employed to evaluate the reliability of the nomogram, as suggested by Coutant [13].

Recently, American Society of Clinical Oncology (ASCO) guidelines for SLNB and ALND have been published, indicating that patients with micro- and macro-metastases may avoid ALND based on the results of IBCSG 23.01 and Z0011 trials [14, 15]. In the prospective randomized IBCSG23-01 trial, only those patients with micro-metastases were randomized to either ALND or no further treatment in patients with positive SLN. The results of this study showed no differences between the two arms both in terms of disease-free and of overall survival. Some challenges, however, still exist regarding this study. Patients accrual stopped prematurely and only 933 out of 1960 patients were enrolled, therefore the study was underpowered. The patient population had a very good prognosis. In fact, sentinel tumor size ≤1 mm was present in 69% of the patients. As a result, the incidence of additional positive NSN in axillary dissection group was 13%, very similar to that found in case of ITCs metastases in SLN. This is also because the author included ITCs in the group of micro-metastases. A strict correlation between the size of micro-metastases (less or greater than 1 mm) and positive NSNs was clearly demonstrated by Rahusen and Viale, respectively [16, 17]. Their results confirm that the presence of 69% of patients with SLN micro-metastases ≤1 mm greatly biases the interpretation in clinical practice. The median follow-up of 5-years is too short to assess the long term incidence of axillary recurrence in this study of this good prognosis group. In NSABP-B6, 20% of nodal recurrences after lumpectomy and 24% of nodal recurrences after ALND and radiotherapy occurred after 5 years [18]. In the IBCSG 23-01 study, the trialists reported that 6681 patients were registered before surgery of which 934 patients were randomized, indicating that only 14% of eligible breast cancer patients met the inclusion criteria for this study and underwent randomization. We anticipate that this may be due to both node negative patients and also patients with multiple positive nodes and other factors, however the breakdown is unknown. The IBCSG 23-01 data supports omission of ALND for the selected group of patients with small, ER+ tumors undergoing breast conservation with planned whole breast radiation. Omitting ALND in SLN positive mastectomy patients and patients undergoing partial breast irradiation requires further investigation. If the primary benefit to these patients primarily through systemic adjuvant therapy and not loco-regional therapy, based on favorable tumor biology, this would seem like the next most logical step. However, data from the NSABP B-32 suggest a statistically significant survival disadvantage after a median follow-up of 8 years for a subset of 611 women with occult nodal disease [19]. Completion axillary dissection had no bearing on this effect and axillary recurrences were equivalent. The 5 year overall survival was 94.6% versus 95.8%, the 5 year disease-free survival was 86.4% versus 89.2% and the 5 year distant disease-free survival was 89.7% versus 92.5%, respectively, all *P* < 0.05. The 8 year median follow-up of the B-32 study is longer than that reported for IBCSG 23-01. It is important to counsel patients that the long-term outcomes of SLN biopsy alone for micro-metastases or ITCs disease are unknown. It is conceivable that based on the IBCSG 23-01 study, patients with favorable tumor characteristics (low T-size, ER+, post menopausal women with low tumor burden in the SLN are potential candidates for limited axillary surgery. In these patients, SLN biopsy can be regarded as a “super selective therapeutic ALND”.

Recently, the recommendation by the ASCO update committee that ALNDs can be safely avoided in patients with one or two SLN metastases undergoing breast conserving surgery with conventional whole-breast radiotherapy (RT) is premature as it is based only on the results of the American College of Surgeons Oncology Group Z11 trial [14, 20]. The shortfalls include the following: recruitment rates were poor (50% of original target); patients recruited into the study had generally low-risk cancers; axillary recurrence was not the predefined primary trial end point; approximately 50% of patients had micro-metastases; a significant proportion of patients had unknown nodal disease. The two groups had slight inequalities in several prognostic characteristics (T stage, grade, lymph-vascular invasion), all favoring the SLN group. Moreover, micro-metastatic-only node disease was present in a statistically significant higher percentage of patients in the SLN group (44.8% *v* 37.5%). A high proportion of patients were lost to follow-up (21% in the ALND group and 17% in the SLN group); there was a significant amount of missing data, and there was no prospective RT quality assurance program to mitigate any bias in RT target volume definition. Recently, Goyal outlined that the most critical issue concerning the generalization of trial is that too many patients with cancers who could have met the eligibility criteria were not represented in the cohort of patients in the trial [21]. Ultimately, the American Society of Clinical Oncology’s recommendation that ALND can be avoided in patients with one or two SLN macrometastases, reflecting the eligibility criteria of Z11, is based on a comparison of 228 patients versus 202 patients which falls significantly short of persuasive based evidence [21]. The perception that the Z0011 trial has not completely convinced the oncological community is demonstrated by the fact that additional trials are still ongoing like POSNOC (Positive Sentinel Node: Adjuvant Therapy Alone Versus Adjuvant Therapy Plus Clearance or Axillary Radiotherapy) trial and the Italian SINODAR-ONE trial that compares SLNB vs. ALND in T1-T2 patients with positive SLN macro-metastases [22]. In regards to the post ACOSOG Z0011 era, another topic still needs to be clarified. Does our new understanding of breast cancer really change clinical practice? Recently, Guth has assessed the potential impact of Z0011 on clinical practice by testing the applicability of its criteria to a European patient population [23]. The author concluded that “the application of Z0011 led to the omission of completing ALND in less than 10% of all SLNB procedures (<6% of all surgically treated BC patients); therefore, we do not think that the perception of Z0011 as “practice-changing” is justified”. In a recent paper, the Results from the Breast Surg ANZ National Breast Cancer Audit database have been questioned for women treated between 2005 and 2010 who would have met the entry criteria for the Z0011 Trial [24]. A total of 64,883 of breast cancer cases were eligible for analysis. 22,731 underwent breast conserving surgery and sentinel node biopsy for invasive breast cancer. A total of 4482 cases (6.9%) fulfilled the criteria for Z-11 Trial. These data seem to point out that many patients do not fulfill the inclusion criteria for Z0011, therefore the nomogram application is still relevant in clinical practice. Other reports also have shown that many patients evaluated with breast cancer may not meet the defined eligibility criteria for avoidance of ALND in the presence of a positive SLN [25, 26]. Reasons may include tumor size, tumor biology, extra-nodal disease, patients undergoing primary chemotherapy, selection of mastectomy, patients treated with PBI or desire to avoid adjuvant breast radiation after breast conserving surgery. Moreover, there are considerations concerning clinical and pathologic subtypes that are less clearly defined in Z0011 trial. For example, patients with lobular histology represented only 7% of the trial population, consequently limiting an accurate analysis of patients with this histologic subtype. Invasive lobular tumors are more likely to have isolated tumor cells in the SLN, reflecting the non-cohesive cellular characteristics that often require IHC detection. [27] and are more likely to have clinical and radiological underestimation of disease burden]. Small-volume nodal disease may have clinical relevance in this patient population unlike those patients with invasive ductal histology. Consideration is therefore given to this difference in biology when we are counseling patients with invasive lobular carcinoma and a positive SLN in performing ALND. Another important factor when making treatment decisions is patient age. Patients older than 18 years of age were eligible to enroll in Z0011. However, the median age of study participants was 54 years in the SLND group and 56 years in the ALND group with more than 62% of patients in each group being older than 50 years. Patient age <50 years was one of only two factors (higher Bloom Richardson grade) associated with local-regional recurrence on the multivariable analysis. There may have been reluctance from the surgeons towards randomizing younger patients with node-positive disease to the SLND-only group and, as a result fewer patients were included in the study population. All these considerations lead to the conclusion that most patients may benefit from OSNA nomograms in the decision-making process on whether to perform ALND or not. At this point, we must consider which OSNA nomogram may have an impact on clinical practice, in other words how many patients with SLN are assessed with OSNA. To the best of our knowledge, there are more than 6000 patients that undergo OSNA positive SLN assessments each year in Europe. Therefore, this certainly justifies the development of the OSNA nomogram that has recently been validated. Moreover, nomogram tools have been shown to decrease the rate of completing axillary dissections in a subset of women with more favorable tumor factors with only a marginally higher recurrence rate (2% *vs.* 0.4% at 23–30 months) [28, 29].

As a surgeon, it is also important to realize that although it may be safe to avoid ALND in an ideal setting in which both adjuvant radiation and systemic therapies are given, in reality not all patients do or plan to complete all the recommended adjuvant therapies, including oral therapies such as tamoxifen, due to the perceived or actual side-effects of these treatments. Further study is needed to improve our understanding of breast tumor biology in order to identify those patients for whom less extensive surgery will not compromise long-term oncologic outcomes. In the mean time, patient counseling for options on low volume axillary disease management should address exactly what data we currently have and what remains unknown. In this context, OSNA nomograms may help surgeons in counseling patients on whether to perform ALND or not and aid patients to accept this decision based on the reliable estimation of the percentage of NSN involvement. Therefore the use of this nomogram achieves two main goals: 1) the choice of the right treatment during the operation, 2) to avoid for the patient a second surgery procedure. The above major results have to be validated in a prospective validation study that is already ongoing.

## Abbreviations

ALND: Axillary lymph node dissection
ASCO: American Society of Clinical Oncology
BC: Breast cancer
ER: Estrogen receptor
LVI: Lymphatic/vascular invasion
NSN: Non sentinel node
OSNA: One step nucleic acid amplification
PgR: Progesterone receptor
ROC: Receiver operating characteristics
RT-LAMP: Reverse transcription loop-mediated isothermal amplification
SLN: Sentinel lymph node
