# Erratum to: Elaboration of a nomogram to predict nonsentinel node status in breast cancer patients with positive sentinel node, intraoperatively assessed with one step nucleic amplification: Retrospective and validation phase

**DOI:** 10.1186/s13046-017-0540-2

**Published:** 2017-05-16

**Authors:** Franco Di Filippo, Simona Di Filippo, Anna Maria Ferrari, Raffaele Antonetti, Alessandro Battaglia, Francesca Becherini, Laia Bernet, Renzo Boldorini, Catherine Bouteille, Simonetta Buglioni, Paolo Burelli, Rafael Cano, Vincenzo Canzonieri, Pierluigi Chiodera, Alfredo Cirilli, Luigi Coppola, Stefano Drago, Luca Di Tommaso, Privato Fenaroli, Roberto Franchini, Andrea Gianatti, Diana Giannarelli, Carmela Giardina, Florence Godey, Massimo M. Grassi, Giuseppe B. Grassi, Siobhan Laws, Samuele Massarut, Giuseppe Naccarato, Maria Iole Natalicchio, Sergio Orefice, Fabrizio Palmieri, Tiziana Perin, Manuela Roncella, Massimo G. Roncalli, Antonio Rulli, Angelo Sidoni, Corrado Tinterri, Maria C. Truglia, Isabella Sperduti

**Affiliations:** 1 Regina Elena National Cancer Institute, Via Elio Chianesi 53, 00144 Rome, Italy; 2 Ospedale di Latina, Latina, Italy; 3 San Camillo, Milan, Italy; 4 Az. Ospedaliera Universitaria Foggia, Foggia, Italy; 5 ASL, Prato, Italy; 6 ULSS 7 Pieve di Soligo, Pieve di Soligo, Italy; 7 Hospital Lluís Alcanyís, Xàtiva, Spain; 8 Università of Piemonte, Vercelli, Italy; 9 Hôpitaux de Lyon, Lyon, France; 10 Hospital Universitario de La Ribera, Alzira, Spain; 11 Centro di Riferimento Oncologico NCI IRCCS - Aviano, Bari, Italy; 12 San Donato, Italy; 13 Policlinico of Bari, Bari, Italy; 14 San Filippo Neri, Florence, Italy; 15 Humanitas Rozzano, Rozzano, Italy; 16 ASST Papa Giovanni XXIII, Bergamo, Italy; 17 Azienda Ospedaliera “Maggiore della Carità” di Novara, Novara, Italy; 18 University of Bari, Bari, Italy; 19 Eugene Marquis Cancer Center, Rennes, France; 20 Humanitas Gavazzeni, Bergamo, Italy; 21 Hampshire Hospitals NHS Foundation Trust, England, UK; 22 University of Pisa, Pisa, Italy; 23 Azienda Ospedaliero-Universitaria OO.RR. Foggia, Foggia, Italy; 24 University of L’Aquila, L’Aquila, Italy; 25 I.R.C.C.S. L. Spallanzani, Rome, Italy; 26 Azienda Ospedaliero-Universitaria Pisana, Pisa, Italy; 27 Humanitas, Pisa, Italy; 28 University of Perugia, Perugia, Italy; 29 USL, Prato, Italy

## Erratum

Upon publication of the original article [1], it was noticed that the affiliation of the authors Vincenzo Canzonieri, Tiziana Perin and Samuele Massarut, “Centro di Riferimento Oncologico NCI IRCCS - Aviano” was incorrectly given as “Centro Regionale Oncologico”. This has now been acknowledged and corrected in this erratum.
